# Hydrocortisone Fails to Abolish NF-κB1 Protein Nuclear Translocation in Deletion Allele Carriers of the *NFKB1* Promoter Polymorphism (-94ins/delATTG) and Is Associated with Increased 30-Day Mortality in Septic Shock

**DOI:** 10.1371/journal.pone.0104953

**Published:** 2014-08-18

**Authors:** Simon T. Schäfer, Sophia Gessner, André Scherag, Katharina Rump, Ulrich H. Frey, Winfried Siffert, Astrid M. Westendorf, Jörg Steinmann, Jürgen Peters, Michael Adamzik

**Affiliations:** 1 Klinik für Anästhesiologie & Intensivmedizin, Universität Duisburg-Essen and Universitätsklinikum Essen, Essen, Germany; 2 Klinische Epidemiologie, Integriertes Forschungs- und Behandlungszentrum (IFB) Sepsis und Sepsisfolgen - Center for Sepsis Control and Care (CSCC), Universitätsklinikum Jena, Jena, Germany; 3 Universität Duisburg-Essen and Universitätsklinikum Essen, Essen Germany; Klinik für Anästhesiologie und Intensivmedizin, Knappschaftskrankenhaus Bochum and Ruhruniversität Bochum, Bochum, Germany; 4 Institut für Pharmakogenetik, Universität Duisburg-Essen and Universitätsklinikum Essen, Essen, Germany; 5 Institut für Medizinische Mikrobiologie. Zurich, Switzerland; D'or Institute of Research and Education, Brazil

## Abstract

**Background:**

Previous investigations and meta-analyses on the effect of glucocorticoids on mortality in septic shock revealed mixed results. This heterogeneity might be evoked by genetic variations. Such candidate is a promoter polymorphism (-94ins/delATTG) of the gene encoding the ubiquitous transcription-factor nuclear-factor-κB (NF-κB) which binds to recognition elements in the promoter of several genes encoding for the innate immune-system. In turn, hydrocortisone inhibits NF-κB nuclear translocation and thus transcription of key immune-response regulators. Accordingly, we tested the hypotheses that hydrocortisone has a *NFKB1* genotype dependent effect on 1) NF-κB1 nuclear translocation evoked by lipopolysaccharide (LPS) in monocytes *in vitro*, and 2) mortality in septic shock.

**Methods:**

Monocytes of volunteers with the homozygous insertion (II; n = 5) or deletion (DD; n = 6) *NFKB1* genotype were incubated with 10 µgml^-1^ LPS ± hydrocortisone (10^-5^M), and NF-κB1 nuclear translocation was assessed (immunofluorescence). Furthermore, we analyzed 30-day-mortality in 160 patients with septic shock stratified for both genotype and hydrocortisone therapy.

**Results:**

Hydrocortisone inhibited LPS induced nuclear translocation of NF-κB1 in II (25%±11;p = 0.0001) but not in DD genotypes (51%±15;p = n.s.). Onehundredandfour of 160 patients with septic shock received hydrocortisone, at the discretion of the intensivist. *NFKB1* deletion allele carriers (ID/DD) receiving hydrocortisone had a much greater 30-day-mortality (57.6%) than II genotypes (24.4%; HR:3.18, 95%-CI:1.61-6.28;p = 0.001). In contrast, 30-day mortality was 22.2% in ID/DD and 25.0% in II genotypes without hydrocortisone therapy. Results were similar when using propensity score matching to account for possible bias in the intensivists' decision to administer hydrocortisone.

**Conclusion:**

Hydrocortisone fails to inhibit LPS induced nuclear NF-κB1 translocation in deletion allele carriers of the *NFKB1* promoter polymorphism (-94ins/delATTG). In septic shock, hydrocortisone treatment is associated with markedly increased 30-day-mortality only in such carriers. Accordingly, previous heterogeneous results regarding the benefit of hydrocortisone in septic shock may be reconciled by genetic variation of the *NFKB1* promoter polymorphism.

## Introduction

Over more than two decades studies have shown both positive and negative effects on mortality of hydrocortisone therapy in septic shock [1]–[3] While Annane et al. reported a decreased 28-day mortality with hydrocortisone therapy [4], others did not find an influence on mortality [1], [2], [5], and even an increased mortality has recently been reported [3]. The uncertainty of the therapeutic value of hydrocortisone therapy in septic shock is also reflected by the current Surviving Sepsis Campaign guidelines, in which hydrocortisone administration in septic shock is recommended with an evidence grade 2C only [6]. However, this wide variability in the effect of hydrocortisone therapy in septic shock could be related to genetic variations.

A candidate gene is the gene encoding the ubiquitous nuclear transcription factor κB (NF-κB1), which binds to recognition elements in the promoter regions of several genes encoding for the innate immune system and induces an inflammatory response [7], [8]. In turn, nuclear translocation of the NF-κB1 protein and thus the induction of key regulators of the innate immune system is inhibited by hydrocortisone [9], [10].

A functional insertion–deletion polymorphism (rs28362491) in the promoter of *NFKB1* (−94ins/del ATTG in relation to the transcription initiation site or −24.219ins/del ATTG in relation to the A as +1 of the initiation codon ATG), which encodes the major isoform of NF-κB1 was found [11]. Furthermore, recently we revealed that this polymorphism is associated with increased *NFKB1* gene expression as well as nuclear translocation of the NF-κB protein following lipopolysaccharide (LPS) stimulation [12]. In addition, this polymorphism is an independent risk factor for 30-day mortality in patients with severe sepsis [12]. In another study, the deletion allele of this *NFKB1* polymorphism was associated with increased illness severity in patients suffering from the acute respiratory distress syndrome [13]. Thus, we could show, that the *NFKB1* promoter polymorphism is functionally active and associated with hyperinflammation [13].

The NF-κB mediated inflammatory response can be inhibited by hydrocortisone via increased IκB expression, direct DNA binding, as well as by altered expression of transcription factors like Jun-C [1], [3], [4], [14]-[17]. Accordingly, there are many reasons to suspect that D allele carriers of the *NFKB1* insertion-deletion (−94ins/delATTG) polymorphism by attenuating the hyperinflammation in septic shock might have a greater benefit from hydrocortisone therapy. Thus, the heterogeneous results regarding hydrocortisone therapy in patients with septic shock might be evoked by or at least associated with this genetic variation.

Accordingly, we tested the hypotheses that the *NFKB1* insertion–deletion (−94ins/delATTG) polymorphism (1) alters nuclear translocation of the NF-κB1 protein in monocytes after lipopolysaccharide with and without hydrocortisone administration, and (2) may be associated with 30-day mortality in patients with septic shock receiving hydrocortisone therapy.

## Materials and Methods

### Ethics statement

This study was reviewed and approved by the Ethics Committee of the Medical faculty of the University of Duisburg-Essen (no. 06-3078) and registered by the German clinical trial database (Deutsches Register für klinische Studien, no.: DRKS00006111). Written informed consent was obtained from all healthy volunteers, and for septic schock patients from the legal guardian of the patient prior to study inclusion. Furthermore, surviving patients were contacted by mail after recovery and where asked whether they object to study participation.

### Dependence of the *NFKB1* promoter polymorphism genotype (−94ins/delATTG) on NF-κB1 nuclear translocation in monocytes following lipopolysaccharide ± hydrocortisone incubation

Venous blood was withdrawn from healthy volunteers having shown to carry the homozygous insertion (II; n = 5) or deletion (DD; n = 6) genotype. Blood samples (Vacutainer CPT tubes, Becton Dickinson, Franklin Lakes, NJ) were centrifuged at 1800 g for 20 minutes using Ficoll density gradient centrifugation tubes, as described [12]. Cells were resuspended using RPMI 1640 medium (Gibco Products Invitrogen Corporation, Grand Island, NY) containing 5% fetal calf serum (Biochrom AG, Berlin, Germany) and antibiotics (100 U ml^-1^ penicillin and 100 µg ml^-1^ streptomycin; Invitrogen Corporation, Carlsbad, CA). The monocyte suspension was transferred into cell culture tubes, and monocytes were allowed to adhere to the surface of the tubes for 2 hours. Then, the supernatant was discarded, fresh RPMI 1640 medium was added, and the cells were allowed to rest for 48 hours (37 °C; 5% CO_2_ in air) prior to the experiments. In the next step, the supernatant was discarded and 500 µl cell suspension (1 x10^6^ monocytes ml^-1^) was added to fibronectin coated (1 mg ml^-1^; Sigma Aldrich, St. Louis, MO) glass plates (12 mm) inside 24-well plates for 24 hours. Cells were incubated with and without 10^-5^ mol hydrocortisone (Bio Reagent H0888, Sigma Aldrich, St. Louis, MO) for 1 hour and then either lipopolysaccharide (LPS, 10 µg ml^-1^, serotype 0111:B4, Sigma Aldrich) or cell culture medium (used as vehicle) were added for another hour. Cells incubated with culture medium only served as controls. Following incubation cells were fixed with ice cold methanol/acetone (1∶1) for 10 minutes at -20 °C.

Immunofluorescence staining was performed using a primary NF-κB1 rabbit anti-human polyclonal p65 antibody (1∶200 dilution, Santa Cruz Biotechnologies, Santa Cruz, CA) followed by a Immunoglobulin G - Alexa-flour 568 coupled goat anti-rabbit antibody (1∶400 dilution, Molecular Probes, Eugene, OR), as described previously [12], [18], [19].

An independent investigator, blind for the treatments and *NFKB1* promoter genotypes, processed all immunofluorescence slides in a randomized order [12]. A Nikon Eclipse E1000 fluorescence microscopy (Nikon GmbH, Düsseldorf, Germany) with NIS-Elements F.30.0 imaging software (Laboratory Imaging, Prague, Czech Republic) was used. Slides were analyzed in a standardized order, and representative images of each quadrant were captured at 20-fold magnification and nuclear NF-κB1 positive cells were counted using an image software (ImageJ, National Institute of Health, Bethesda, MD) [12], [18].

### 30-day mortality in patients with septic shock when stratified for *NFKB1* promoter polymorphism genotype (−94ins/delATTG) and hydrocortisone therapy

Patients were eligible for the study when they fulfilled the criteria for septic shock as defined [1]-[3], [20], and then were prospectively included in this observational trial. Primary study endpoint was genotype dependent 30-day mortality. Between 2010 and January 2014, 160 patients (101 males, 59 females, mean age: 57 years ± 16) admitted to our intensive care unit were prospectively enrolled. Information on hydrocortisone treatment was available for all patients and this was retrospectively added to the database. Patients which met the exclusion criteria, i.e., those with an age less than 18 years, those of non-Caucasian ethnicity, or refusal of study participation were excluded. Thus, all patients were white Germans of Caucasian ethnicity.

Clinical and demographic data upon study entry (table 1) including Simplified Acute Physiology Score II (SAPS II) [4], [21], [22] and the Sequential Organ Failure Assessment score (SOFA) [1], [2], [5], [23]–[25] were calculated over the first 24 hours after the patient met inclusion criteria, and all patients were followed for up to 30 days. Patients were treated with a multimodal concept which included protective mechanical ventilation, hemodynamic, antibiotic, and diagnostic management, as published previously [3], [12], [18]. Continuous hemofiltration/dialysis was technically performed by the Department of Nephrology according to standardized protocols.

**Table 1:** Characteristics of septic shock patients with the II and ID/DD genotype of the *NFKB1* promoter polymorphism (−94ins/delATTG) when stratified according to hydrocortisone therapy.

	II genotype	n = 65	ID/DD genotype	n = 95	
Hydrocortisone therapy	+ n = 45	- n = 20	+ n = 59	- n = 36	p-value
Characteristics^a^					
Median age^f^ [years] (IQR^g^)	57 (47-66)	74 (56-76)	56 (49-66)	58 (44-68)	0.020
Females/males^f^; N (%)	12/33 (27/73)	7/13 (35/65)	25/34 (42/58)	15/21(42/58)	0.356
Median height [m] (IQR^g^)	1.76 (1.70-1.84)	1.69 (1.63-1.79)	1.72 (1.63-1.80)	1.70 (1.60-1.80)	0.788
Median body weight [kg] (IQR)	82 (74-100)	72 (65-81)	82 (65-98)	74 (56-88)	0.066
Median body-mass-index [kg/m^2^] (IQR^g^)	26 (24-31)	24 (23-26)	26 (24-33)	25 (22-28)	0.124
Median arterial pressure^f^ [mmHg] (IQR^g^)	80 (70-90)	78 (73-92)	80 (73-94)	81 (70-90)	0.544
Median noradrenaline^f^ dosage [mg h^-1^] (IQR^g^)	0.2 (0.0-0.5)	0.1 (0.0-0.7)	0.3 (0.1-1.8)	0.1 (0.0-0.8)	0.137
Median creatinin serum concentration^f^ [mg dl^-1^] (IQR)	1.6 (1.0-2.4)	1.5 (0.8-2.2)	1.9 (1.3-2.6)	1.4 (0.9-2.6)	0.128
Dialysis^f^ yes/no; N (%)	25/17 (41/59)	8/10 (44/56)	44/15 (75/25)	18/15 (55/45)	0.068
Median SAPS II^f^ (IQR^g^)	51 (34-63)	49 (35-62)	43 (33-57)	43 (33-53)	0.615
Median SOFA (IQR^g^)	12 (10-15)	11 (9-13)	14 (10-17)	11 (10-13)	0.059
Infection^b^					
Median pro-calcitonin^f^ concentration [µgl^-1^] (IQR^g^)	5 (1-24)	4 (2-6)	8 (3-18)	2 (1-9)	0.002
Median C-reactive protein^f^ concentration [gl^-1^] (IQR^g^)	14 (6-25)	18 (11-25)	14 (7-21)	12 (5-16)	0.454
Median leukocyte concentration^f^ [nl^-1^] (IQR^g^)	15 (11-20)	17 (11-20)	15 (9-24)	11 (8-17)	0.225
Primary diagnoses; N (row%)^c^					
Cardiovascular	10 (34)	7 (24)	3 (10)	9 (31)	^e^
Hematooncological	2 (29)	0 (0)	1 (14)	4 (57)	
Abdominal	17 (26)	4 (6)	36 (54)	9 (14)	
Pulmonary	10 (31)	2 (6)	13 (41)	7 (21)	
Renal	1 (14)	2 (29)	3 (43)	1 (14)	
Other	5 (29)	4 (24)	2 (12)	6 (35)	
Blood cultures; N (row%)^c^					^e^
Grampositive isolates only	8 (33)	3 (13	9 (38)	4 (17)	
Gramnegative isolates only	4 (19)	2 (10)	8 (38)	4 (17)	
Fungal isolates only	2 (50)	0 (0)	2 (50)	0 (0)	
Mixed isolates	12 (32)	5 (13)	11 (29)	10 (26)	
Negative blood cultures	18 (28)	9 (14)	23 (36)	14 (22)	

II  =  homozygous NF*h*κB insertion genotype; ID  =  heterozygous deletion genotype.

DD  =  homozygous deletion genotype; SAPS II  =  Simplified Acute Physiology Score II.

SOFA  =  Sequential Organ Failure Assessment.

a total N of missing values for each variable (in order of the variables): 0, 0, 30, 29, 31, 0, 0, 9, 8, 0, 49.

b total N of missing values for each variable (in order of the variables): 15, 15, 4.

c total N of missing values for each variable: 2, 9.

d Kruskal-Wallis-Test for continuous data or generalized Fishers' exact Test for count data.

e no statistical test has been applied here.

f variables used for the propensity score construction.

g IQR: interquartile range.

DNA was isolated from patients and genotyped for the *NFKB1* promoter polymorphism (−94ins/delATTG), as described below [6], [12], [13], [26].

### DNA genotyping

Patients genomic DNA was extracted from leukocytes extracted from whole blood using a standard method as described previously (QIAamp, Qiagen, Hilden, Germany) [7], [8], [12]. *NFKB1* insertion/deletion genotypes were determined by pyrosequencing [9], [10], [12]. A 200bp PCR fragment was amplified using specific primer (primer *NFKB1*_del/ins_f(5′-ATGGACCGCATGACTCTATCAG-3′) and biotinylated primer *NFKB1*_del/ins_BIO_r(5′-GGGGCGCGCGTTAGGCGG-3′)). PCR reaction was operated at an annealing temperature of 60°C using a 50 µl reaction mixture containing a commercially available PCR master mix (Eppendorf, Hamburg, Germany). A PSQ96 MA (Pyrosequencing, Uppsala, Sweden) PCR machine was used for pyrosequencing using sequencing primer *NFKB1*_del/ins_seq (5′-CGTTCCCCGACCAT-3′). Following institutional quality requirements, genotype results were confirmed for randomly chosen samples using a different nucleotide injection order, as described [11], [12].

### Statistical analyses

**Figure 1:**
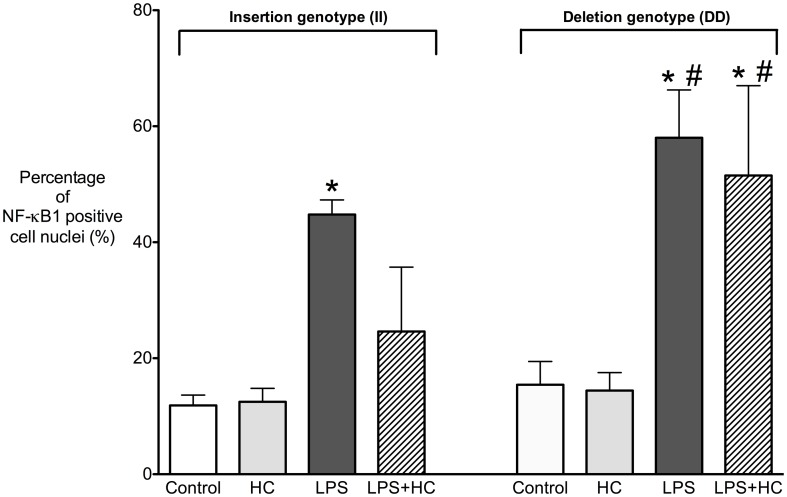
Percentage of NF-κB1 positive monocytes according to the *NFKB1* promoter polymorphism (-94ins/delATTG) following lipopolysaccharide (LPS) incubation with and without hydrocortisone (HC). Hydrocortisone significantly decreased the percentage of NF-κB1 positive cell nuclei in LPS stimulated monocytes of II genotype individuals, but not in those of the DD genotype individuals, which showed an unchanged high percentage of NF-κB1 positive cell nuclei. There was no difference in the percentage of NF-κB1 positive cells both in unstimulated and hydrocortisone (HC) treated cells. * p<0.0001 vs. control; # p<0.01 vs. II genotype. II  =  homozygous insertion genotype; DD  =  homozygous deletion genotype.

Results of the *in vitro* cell studies are reported by bar plots (mean ± standard deviation (SD); Figure 1) and were assessed by ANOVA with two factors (genotype and hydrocortisone therapy) due the normally distributed percentage of nuclear-NF*h*κB positive cells. A post-hoc two-tailed Student's t-test for independent samples was performed applying a Bonferroni correction for multiple tests. Analyses were performed using the Graphpad Prism 5 software (San Diego, CA) [12], [18].

Clinicopathologic characteristics of the 160 patients with septic shock are presented in Table 1 when stratified according to the four subgroups (*NFKB1* genotype x hydrocortisone therapy). The *NFKB1* promoter polymorphism genotype distribution was tested for deviations from the Hardy Weinberg equilibrium (exact two-sided p-value 1.00).

**Figure 2:**
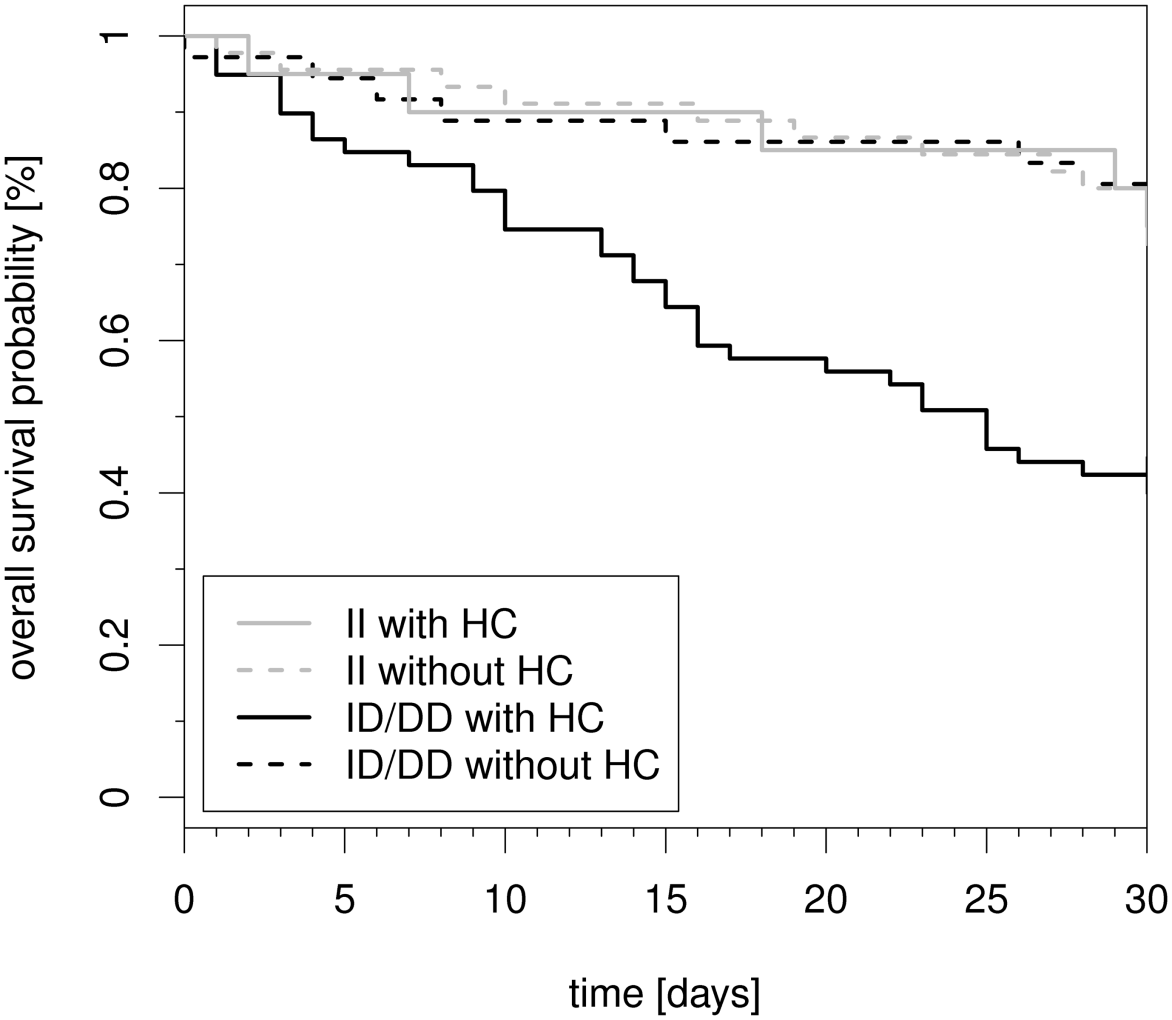
Kaplan–Meier plot of 30-day mortality of patients with septic shock stratified both by *NFKB1* promoter polymorphism (−94ins/delATTG) and hydrocortisone therapy. Kaplan-Meier estimators for the four subgroups for all 160 septic shock patients. DD  =  homozygous deletion genotype. ID  =  heterozygous deletion genotype. II  =  homozygous insertion genotype.

We investigated associations between the clinicopathologic characteristics, *NFKB1* promoter polymorphism genotype, and hydrocortisone therapy with overall 30-day survival, defined as the interval from time of diagnosis of septic shock until death. Patients alive after the 30-day follow-up were regarded as censored. Kaplan-Meier estimators were used to display the overall 30-day survival data in the respective four subgroups followed by log-rank tests to compare the subgroups (Figure 2). Table 2 displays hazard ratio (HR) point estimates, 95% confidence intervals (abbreviated 95% CI), and p-values derived from Cox regression models. Multivariate analyses included two steps with a focus on the four subgroups of interest.

**Table 2:** Univariate and multivariable associations to 30-day overall survival in 160 septic shock patients.

			Univariate Cox models		Multivariable Cox Model 1^c (main effects model; N = 152)^		Multivariable Cox Model 2^d (“interaction” model; N = 152)^	
Prognostic Variable^a^	Units	N^b^	HR (95%CI)	p-value	HR (95%CI)	p-value	HR (95%CI)	p-value
*NFKB1* promoter polymorphism genotype	II	65	1	0.03	1	0.003		
	ID/DD	95	1.91 (1.08;3.36)		2.59 (1.39;4.82)			
Hydrocortison therapy	without HC	56 104	1	0.02	1	0.005		
	with HC		2.15 (1.16;3.98)		2.64 (1.33;5.23)			
*NFKB1* promoter polymorphism genotype and HC therapy	II with HC	45	1	<0.0001^e^			1	<0.0001^e^
	II without HC	20	1.03 (0.36;2.97)				0.74 (0.20;2.70)	
	ID/DD with HC	59	3.18 (1.61;6.28)				3.52 (1.72;7.22)	
	ID/DD without HC	36	0.92 (0.37;2.97)				1.06 (0.42;2.70)	
Age^a^	per 5 years	160	1.00 (0.92;1.08)	0.92				
	< 57 years	77	1	0.53				
	≥ 57 years	83	0.85 (0.51;1.42)					
Sex	Female	59	1	0.21				
	Male	101	0.71 (0.43;1.20)					
Height	per cm	130	1.00 (0.98;1.03)	0.92				
Body weight	per kg	131	1.00 (0.99;1.02)	0.49				
Body-mass-index	per kg/m^2^	129	1.01 (0.97;1.06)	0.55				
Arterial pressure	per mmHg	160	0.98 (0.97;1.00)	0.12	0.98 (0.96;1.00)	0.09	0.98 (0.96;1.00)	0.07
Noradrenaline dosage	per mg/h	160	1.01 (0.97;1.06)	0.67				
Creatinin serum- concentration	per mg/dl	151	1.14 (0.89;1.47)	0.29				
Dialysis	no	57	1	0.09	1	0.51	1	0.54
	Yes	95	1.65 (0.92;2.95)		1.22 (0.67;2.23)		1.21 (0.66;2.20)	
SAPS II	per point	160	1.01 (1.00;1.03)	0.18				
SOFA	per point	111	1.06 (0.98;1.15)	0.15				
Pro-calcitonin concentration	per µg/l	145	1.00 (1.00;1.01)	0.26				
C-reactive protein concentration	per g/l	145	1.01 (0.98;1.03)	0.56				
Blood leukocyte concentration	per n/l	156	1.00 (0.98;1.03)	0.88				

a Multivariable Cox regression analysis for continuous prognostic variables are displayed for the continuous linear predictor; for “Age” we performed several sensitivity analyses including those displayed, in addition to a combined linear and quadratic continuous predictor – these transformations had no impact on the conclusions.

b the number of available data for a particular variable in the univariate analysis.

c model in which all main effects of potential prognostic factors with univariate uncorrected p-values < 0.15 are included.

d like model 1 (see c) but main effects of *NFKB1* genotype and hydrocortisone therapy are included as four subgroup.

In model 1 all main effects with univariate p-values less than 0.15 were investigated simultaneously (Table 2). To address potential interactions of genotype and hydrocortisone therapy, we developed a model 2 in which we modeled four subgroups separately, in addition to the same main effects of model 1 (Table 2). Furthermore, we also performed automatic forward and backward selection strategies, and model diagnostics including graphical and formal checks.

**Figure 3:**
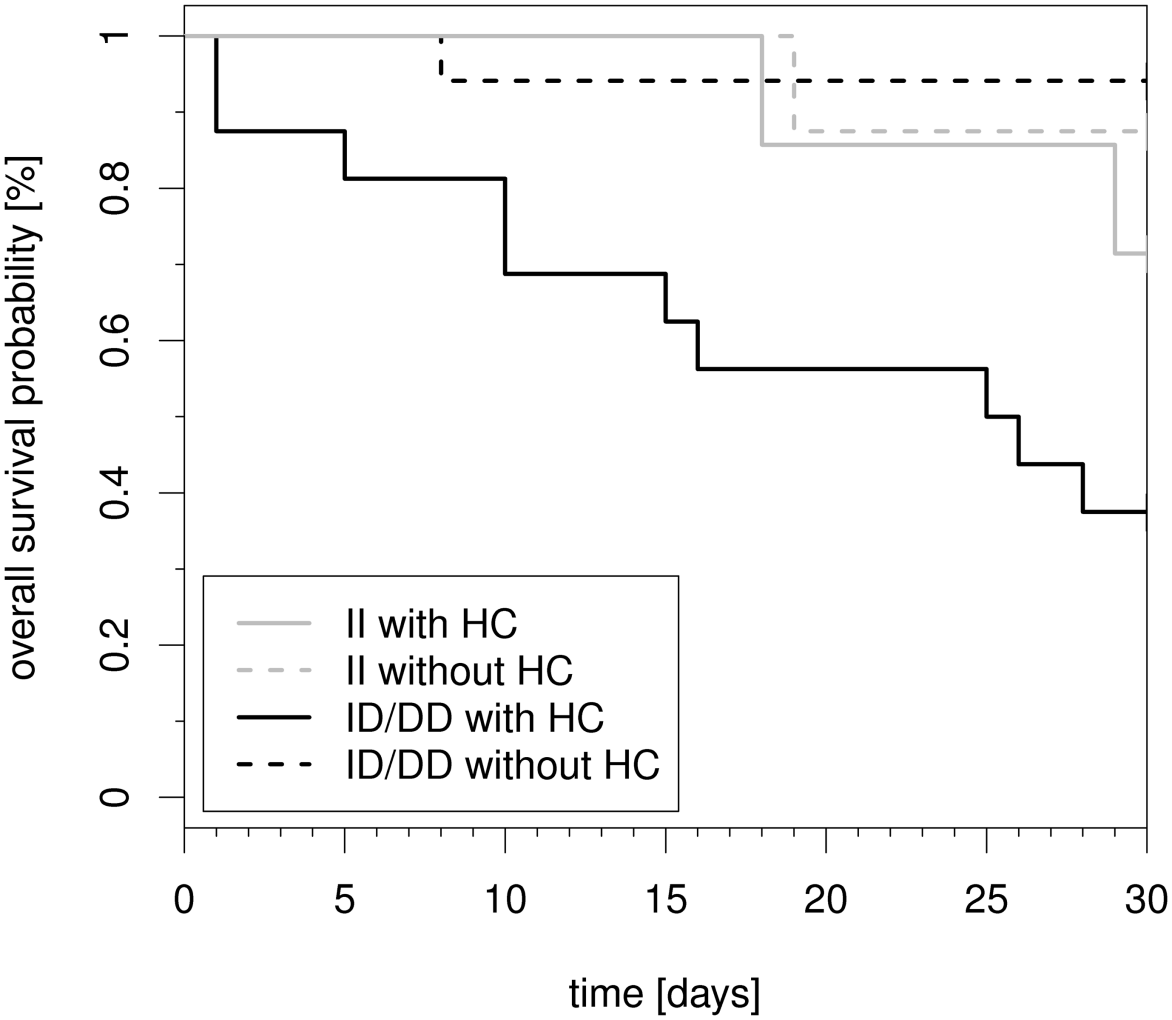
Kaplan–Meier plot of 30-day mortality of patients with septic shock stratified both by *NFKB1* promoter polymorphism (−94ins/delATTG) and hydrocortisone therapy. Kaplan-Meier estimators for the four subgroups in 2x24 matched patients of the propensity score analysis. DD  =  homozygous deletion genotype. ID  =  heterozygous deletion genotype. II  =  homozygous insertion genotype.

Finally, to address the potential dependency of hydrocortisone therapy initiation on clinicopathologic characteristics, we also applied propensity score matching (Figure 3). The propensity score for the probability of hydrocortisone therapy initiation was based on the variables highlighted in Table 1. We followed the recent recommendations on propensity score analyses for survival data [12], [27] and provide details on the analysis as Figure S1. Finally, as part of our sensitivity analyses, we also performed all time-to-event analyses for a dichotomized 30-day mortality outcome.

Statistical analyses were performed using SPSS 21 (SPSS Inc, Chicago, IL) and R 3.0.2 (http://www.R-project.org). All reported p-values are nominal, two-sided, and we applied a significance level of 5%.

## Results

### Nuclear translocation of NF-κB1 in monocytes according to the *NFKB1* promoter polymorphism genotype (−94ins/delATTG)

LPS increased the percentage of NF-κB1 positive cell nuclei both in the II genotype (control: 12% ± 2 vs. LPS: 45% ± 3; p<0.0001; Figure 1) and in the DD genotype (control: 15% ± 4 vs. LPS: 58% ± 9; p<0.0001), with the effect being more pronounced in DD genotype individuals (p<0.01 II vs. DD genotype).

More important, hydrocortisone decreased the LPS induced NF-κB1 nuclear translocation in II genotypes (LPS + hydrocortison: 25% ± 11) but failed to inhibit NF-κB1 nuclear translocation in DD genotypes (52% ± 15%; p<0.01). Thus, following hydrocortisone the DD genotype is associated with a persistently high NF-κB nuclear translocation in response to LPS. In contrast, we observed no evidence for differences in the percentage of NF-κB positive cells between unstimulated and hydrocortisone treated cells (all p>0.05).

### 30-day mortality in patients with septic shock when stratified by *NFKB1* promoter polymorphism genotype (−94ins/delATTG) and hydrocortisone therapy

30-day mortality markedly differed between the four cohorts as defined by the *NFKB1* insertion/deletion (−94ins/delATTG) polymorphism genotype status and administration or not of hydrocortisone (p<0.0001; Figure 2). Onehundredandfour of 160 patients received hydrocortisone therapy at the discretion of the intensivist in charge. In the crude analysis which included all patients and ignored possible confounders the estimated 30-day mortality was 57.6% (34/60 patients) for combined ID/DD genotypes with hydrocortisone therapy but was only 24.4% (11/45 patients) in II genotype individuals with hydrocortisone therapy (p<0.0001 for the comparison of these two subgroups). Furthermore, 30-day mortality was 22.2% (8/36) in ID/DD genotypes without hydrocortisone and 25.0% (5/20 patients) in II genotypes without hydrocortisone therapy.

Results obtained from the propensity score matching were similar to those obtained from the crude model (p = 0.001, Figure 3) although they were based on a smaller sample of 2x24 patients matched for hydrocortisone therapy. In addition, sensitivity analyses for a dichotomized 30-day mortality outcome were performed, and led to the same conclusions (data not shown).

### Cox regression analysis

The results of the univariate and multivariable Cox analyses are displayed in table 2. The univariate analysis revealed that both the *NFKB1* polymorphism genotype status (HR for the ID/DD genotype: 1.91, 95% CI (1.08;3.36), p = 0.03) and administered hydrocortisone therapy (HR: 2.15, 95% CI (1.16;3.98), p = 0.02) were prognostic factors for 30-day survival. However, the combination of both ID/DD genotype and administered hydrocortisone therapy showed the largest impact (HR: 3.18, 95% CI (1.61;6.28), p = 0.001). Formally, this is also underlined by an interaction term (p = 0.10) in a model with genotype and hydrocortisone therapy main effects and their interaction. Our results remained robust in the multivariate analyses (model 1 and 2; table 2) in which arterial pressure and dialysis were additionally considered and in sensitivity analyses with automatic forward and backward variable selection (data not shown).

### Characteristics of patients with septic shock stratified by *NFKB1* promoter polymorphism genotype (−94ins/delATTG) and hydrocortisone therapy

All patients had severe sepsis with septic shock and were mechanically ventilated at the time of study inclusion. Detailed characteristics of the patients are shown in Table 1 when stratified by the four subgroups based on the combination of *NFKB1* genotype and hydrocortisone therapy. We observed some evidence for subgroup differences (explorative p-values ≤ 0.05) for age (slightly higher age for patients with insertion genotype who were not treated with hydrocortisone) and pro-calcitonin concentrations (higher in hydrocortisone treated patients). For other important patients characteristics no evidence for any cohort differences was observable. In particular, no differences were seen between subgroups for the severity of illness, as assessed by SAPS II and SOFA.

## Discussion

Our study shows, that a common genetic variation in the promoter of *NFKB1* (insertion–deletion polymorphism −94ins/delATTG) contributes to the outcome of patients with septic shock treated with hydrocortisone. Of note, hydrocortisone treatment in deletion allele carriers is 1) a strong and independent predictor for 30-day mortality and 2) fails to inhibit the lipopolysaccharide induced nuclear translocation of NF-κB1 in monocytes. This may reconcile the previous heterogeneous results regarding the benefit of hydrocortisone administration in septic shock.

Hydrocortisone therapy in D allele carriers of the *NFKB1* insertion/deletion (−94ins/delATTG) polymorphism was a strong and independent prognostic factor for 30-day mortality. Thus, D allele carriers undergoing hydrocortisone therapy in septic shock had a more than 3-fold increased risk for death compared to D allele carriers without hydrocortisone therapy as well as the homozygous insertion genotype independent of hydrocortisone therapy. Our observation remained robust in multivariate regression analysis when controlling for other potential prognostic factors. Since one might assume that the observed effect might be due to an a priori bias on part of the intensivist assigning or not a patient to hydrocortisone therapy, we also analysed our data using the propensity score matching methodology [13], [27]. Again, 30-day mortality was worst in septic shock patients who received hydrocortisone therapy and who carried the ID/DD genotype. Thus, our data show that the heterogeneous results regarding corticoid therapy in septic shock might in part be due to the genetic variation -94ins/delATTG in the promoter of *NFKB1*.

Our study also revealed that hydrocortisone had a *NFKB1* genotype dependent effect on monocytes in vitro. In the present study, whole blood from healthy donors was incubated with LPS in vitro to investigate any effects of the *NFKB1* promoter genotype when cells were additionally exposed to hydrocortisone. Previously, we had already demonstrated that the D allele of the *NFKB1* insertion/deletion (−94ins/delATTG) polymorphism is associated with increased nuclear translocation of the NF-κB1 protein and an increased mortality in septic patients [12], [13]. Thus, deletion allele carriers could benefit from hydrocortisone therapy by decreasing the present hyperinflammation.

Surprisingly, our experiments showed in deletion allele carriers that hydrocortisone administration failed to decrease the LPS evoked increased nuclear translocation of the NF-κB1 protein. In contrast, in II genotype individuals, hydrocortisone was a potent inhibitor of the nuclear translocation of NF-κB1. Hence the DD genotype is characterized by an unsuppressed inflammatory response following LPS and hydrocortisone incubation, whereas the percentage of NF-κB1 positive cell was decreased in the II genotype with LPS and hydrocortisone administration and thus did not differ from controls. In addition, deletion allele carriers with septic shock undergoing hydrocortisone therapy had a more than 3-fold increased mortality, whereas mortality in deletion allele carriers without hydrocortisone did not differ from II genotype individuals. The molecular mechanisms involved in the abolition of hydrocortisone's effect and the increased mortality in D allele carriers undergoing hydrocortisone therapy remain unclear. However, it is well known that a functional balance between inflammation and anti-inflammation is crucial in surviving septic shock [1], [3], [4], [14]-[17], [28]. Thus, we can only speculate that D allele carriers are characterized not only by an initial hyperinflammation [12] but also by an increased anti-inflammatory response, which could have been increased even further by hydrocortisone administration. While hydrocortisone did not decrease nuclear translocation of NF-κB1 in D allele carriers, other hydrocortisone related pathways like direct DNA binding or anti-inflammatory transcription factor concentrations might be increased or altered by hydrocortisone [1], [12], [14], [17]–[19].

Although our study cannot pinpoint the underlying mechanisms, we show that the heterogeneous results regarding the potential benefit of hydrocortisone therapy in septic shock may be explained by this genetic variation. Accordingly, if possible, retrospective subcohort analysis stratified for the NFKB1 genotype (-94ins/delATTG) should be performed on the data originating from the large randomized trials on hydrocortisone therapy in septic shock [3], [4], [12]. It is only then, that it can finally be assessed whether hydrocortisone therapy is beneficial or harmful in septic shock. Furthermore, this might precipitate a first step towards personalized medicine also in the field of sepsis and septic shock where it might depend on the genes whether a therapy evokes harm or benefit. In any case, our data show that hydrocortisone therapy in septic shock, dependent on the *NFKB1* genotype, is not innocuous at all and can increase the risk of death.

Our study has several limitations. The sample size of 160 patients with septic shock is rather small and our results require independent replication in larger cohorts. However, consenting patients with septic shock patients are hard to enroll given that only about 2-20% of all intensive care unit patients suffer from severe sepsis including septic shock [1], [12], [18], [29], [30].

Despite having used even two different statistical approaches for analysis, unidentified confounders or an unknown selection bias, inherent to all observational studies, cannot be excluded. However, all septic patients were treated with a standardized multimodal regimen including withdrawal of blood cultures and antimicrobial therapy [6]. Furthermore, we observed no evidence for subcohort differences regarding serum creatinine concentration, SAPS II, SOFA score distributions, arterial blood pressure or noradrenaline dosage. Furthermore, while all patient data were acquired prospectively, hydrocortisone therapy was not an a *priori* focus of the study. However, exact information about hydrocortisone therapy was obtained for all patients and this information was added to the database retrospectively. Anyhow, our data show that hydrocortisone therapy in D allele carriers is an important and independent risk factor for 30-day mortality in septic shock. Finally, although we could show that hydrocortisone fails to inhibit nuclear translocation of NF*h*κB1 in monocytes, the underlying molecular mechanism cannot be explained.

In conclusion, hydrocortisone therapy in deletion allele carriers of the *NFKB1* promoter polymorphism (-94ins/delATTG) is a strong and independent predictor for 30-day mortality of septic shock. Furthermore, hydrocortisone administration in deletion allele carriers fails to inhibit lipopolysaccharide induced NF-κB1 nuclear translocation. Accordingly, the heterogeneous results regarding the benefit of hydrocortisone in septic shock may be explained and reconciled by genetic variation.

## Supporting Information

Figure S1 We applied propensity score matching using the R-package MatchIt (Institute for statistics and mathematics; University Wien, Austria). We modeled the probability of hydrocortisone therapy initiation using the variables age, sex, arterial pressure, noradrenaline dosage, creatinin serum-concentration, dialysis, SAPS II, pro-calcitonin concentration, C-reactive protein concentration, and leukocyte concentration (i.e., we focused on variables with no or limited missing values). The variables were scaled as described in table 1. We used nearest neighbour caliper matching for a caliper distance defined by 0.25 times the standard deviation of the propensity scores. Figure S1) shows the histograms of the propensity score distributions of the 2x24 matched patients.
**Histograms of the propensity score distributions of the matched patients.** The histogram of the 24 patients without hydrocortisone therapy initiation is superimposed by the histogram of the 24 patients with hydrocortisone therapy initiation.
(TIFF)
