# Correction: Hydrocortisone Fails to Abolish NF-κB1 Protein Nuclear Translocation in Deletion Allele Carriers of the *NFKB1* Promoter Polymorphism (-94ins/delATTG) and Is Associated with Increased 30-Day Mortality in Septic Shock

**DOI:** 10.1371/journal.pone.0112369

**Published:** 2014-10-27

**Authors:** The PLOS ONE Staff

The affiliation for the seventh and eighth authors is incorrect. Astrid M. Westendorf and Jörg Steinmann are both affiliated with: Institut für Medizinische Mikrobiologie, University Duisburg-Essen and Universitätsklinikum Essen, Essen, Germany
