# Supplementary figures and images for: Hydrocortisone Fails to Abolish NF-κB1 Protein Nuclear Translocation in Deletion Allele Carriers of the NFKB1 Promoter Polymorphism (-94ins/delATTG) and Is Associated with Increased 30-Day Mortality in Septic Shock
Source: PLoS One. 2014 Aug 18;9(8):e104953. doi: 10.1371/journal.pone.0104953 (PMC4136840; doi:10.1371/journal.pone.0104953)

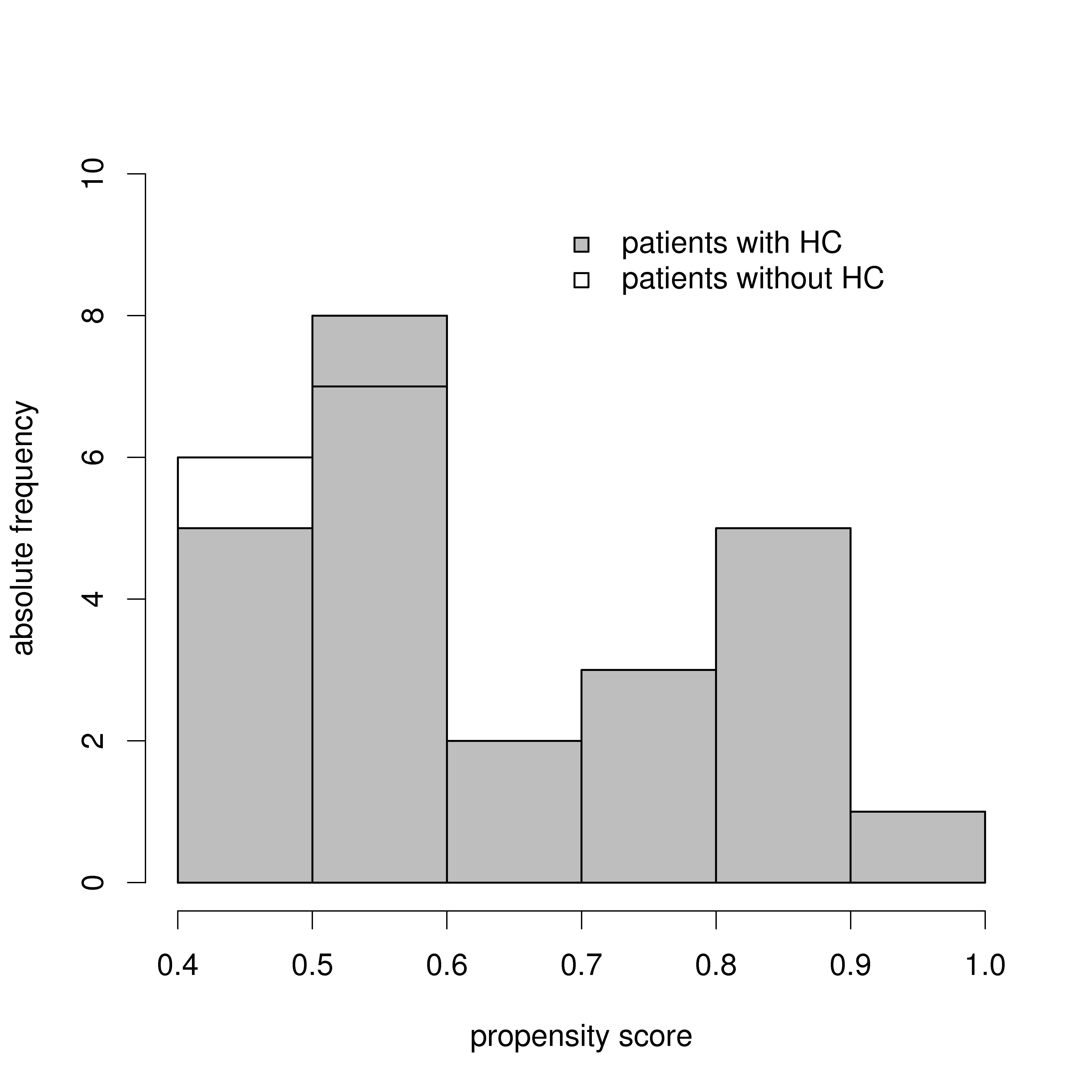

Supplement: Figure S1 — We applied propensity score matching using the R-package MatchIt (Institute for statistics and mathematics; University Wien, Austria). We modeled the probability of hydrocortisone therapy initiation using the variables age, sex, arterial pressure, noradrenaline dosage, creatinin serum-concentration, dialysis, SAPS II, pro-calcitonin concentration, C-reactive protein concentration, and leukocyte concentration (i.e., we focused on variables with no or limited missing values). The variables were scaled as described in table 1. We used nearest neighbour caliper matching for a caliper distance defined by 0.25 times the standard deviation of the propensity scores. Figure S1) shows the histograms of the propensity score distributions of the 2x24 matched patients. Histograms of the propensity score distributions of the matched patients. The histogram of the 24 patients without hydrocortisone therapy initiation is superimposed by the histogram of the 24 patients with hydrocortisone therapy initiation. (TIFF) [file pone.0104953.s001.tiff]
